# Optimized immunofluorescence for liver structure analysis: Enhancing 3D resolution and minimizing tissue autofluorescence

**DOI:** 10.1093/biomethods/bpaf023

**Published:** 2025-03-26

**Authors:** Elena Zoppolato, Hasse Mol, Carlos Estrella-García, Nicole Vizcaino-Rodríguez, Diana Sanchez, Nicole Procel, Isabel Baroja, Leticia Sansores‐Garcia, Iván M Moya

**Affiliations:** VIB Center for Cancer Biology and KU Leuven Department of Oncology, KU Leuven, Leuven, 3000, Belgium; VIB Center for Cancer Biology and KU Leuven Department of Oncology, KU Leuven, Leuven, 3000, Belgium; Cancer Research Group, School of Biotechnology, Faculty of Engineering and Applied Sciences, Universidad de Las Américas, Quito, 170124, Ecuador; Cancer Research Group, School of Biotechnology, Faculty of Engineering and Applied Sciences, Universidad de Las Américas, Quito, 170124, Ecuador; Cancer Research Group, School of Biotechnology, Faculty of Engineering and Applied Sciences, Universidad de Las Américas, Quito, 170124, Ecuador; Cancer Research Group, School of Biotechnology, Faculty of Engineering and Applied Sciences, Universidad de Las Américas, Quito, 170124, Ecuador; Cancer Research Group, School of Biotechnology, Faculty of Engineering and Applied Sciences, Universidad de Las Américas, Quito, 170124, Ecuador; VIB Center for Cancer Biology and KU Leuven Department of Oncology, KU Leuven, Leuven, 3000, Belgium; Cancer Research Group, School of Biotechnology, Faculty of Engineering and Applied Sciences, Universidad de Las Américas, Quito, 170124, Ecuador

**Keywords:** Liver, Tissue autofluorescence, 3D structures

## Abstract

The study of liver biology and pathology through marker expression analysis and tissue structure visualization is constrained by the high autofluorescence caused by the presence of lipofuscins, vitamin A, and lipid droplets, which traditional staining methods do not effectively quench. This leads to low signal-to-noise ratios, obscured expression levels, and reduced structural resolution. We mitigated liver tissue autofluorescence using Sudan Black B staining, which effectively quenches background signals from lipid and lipofuscin accumulation. Additionally, these protocols typically use thin paraffin sections (5–7 µm), which limit the analysis of larger and more complex liver structures. Liver tissue is highly organized in three dimensions, with large hepatocytes (20–30 µm in diameter) arranged around sinusoids and bile canaliculi, which form intricate branching networks. Thin sections cannot capture this 3D organization, providing only a “snapshot” of the tissue at one plane. Here, we present an optimized immunofluorescence protocol using 100–200 µm vibratome-cut liver sections to enable a more comprehensive 3D-like analysis of liver architecture. Finally, our protocol includes antigen retrieval steps tailored to each antibody, maximizing epitope accessibility and signal clarity. Together, these improvements provide a robust method for detailed liver studies with enhanced specificity and structural resolution in immunofluorescent staining. This protocol is particularly suited for researchers focused on liver regeneration, cancer, chronic disease pathology, and structural analysis. However, other researchers interested in exploring complex tissue structures in other autofluorescent tissues, such as the kidney, brain, pancreas, spleen, and adipose tissue, will also find this method beneficial.

## Introduction

The liver is a complex organ with critical functions in metabolism, detoxification, and regeneration. Understanding its structure and pathology is essential for addressing diseases such as liver cancer, fibrosis, metabolic associated steatotic liver disease (MASLD) and cholangiopathies. Immunofluorescence staining (IF) is a widely used technique to study protein expression and tissue organization. However, liver tissue presents unique challenges due to high autofluorescence caused by lipofuscin, vitamin A, and lipid droplets [1–3]. These compounds can obscure fluorescent signals and compromise the clarity of the data. Tissue autofluorescence emits light under common excitation wavelengths, often overwhelming specific antibody signals and obscuring target protein detection. This compromises the accuracy of IF analysis, leading researchers to use chromogenic detection methods not affected by background fluorescence instead. However, chromogenic methods lack the spatial and intensity resolution that IF can offer, limiting detailed studies on protein expression patterns in liver tissues. Additionally, IF enables the simultaneous detection of multiple markers and their colocalization within the same tissue section—capabilities unavailable in chromogenic methods. Thus, we developed this protocol with a notable improvement, the use of Sudan Black B to quench liver tissue autofluorescence, to study liver cancer and regeneration [4, 5].

Standard paraffin-embedded sections are typically around 5 µm thick, which is insufficient to capture the 3D architecture of the liver [6]. To fully appreciate the complex and branching structures of the liver, including sinusoids, hepatocyte plates, and bile canaliculi, thicker tissue sections are needed. Given that hepatocytes can be 20–30 µm in diameter, with entire structural networks extending over 100 µm, thicker sections—ranging from 100 to 200 µm—provide a more comprehensive view. These thick sections preserve the continuity of sinusoidal and canalicular networks, enabling a more accurate assessment of spatial relationships within liver microarchitecture, which is especially critical for studies focused on liver structure and pathology.

Common fixatives, like formalin, cause protein cross-linking, which can alter epitopes and reduce antibody binding efficiency [7, 8]. This is a critical yet often overlooked issue, often forcing researchers to test multiple antibodies in hopes of finding one that reliably recognizes the protein of interest. Antigen retrieval steps are crucial for unmasking epitopes that may be hidden or altered due to tissue fixation. Here, we include different antigen retrieval techniques, which can be specifically tailored for each antibody to be used in IF. By incorporating antigen retrieval methods based on the use of denaturing buffers such as Sodium Citrate or Tris-EDTA, this protocol ensures improved epitope accessibility, enhancing the signal quality of various antibodies. This adjustment significantly broadens the range of antibodies that can be effectively used in IF staining of liver tissues.

In summary, this protocol provides a streamlined approach to liver IF by addressing major obstacles, such as autofluorescence, 3D structural visualization, and optimized antigen retrieval. Although simple, these adjustments make IF a more viable and informative technique for liver research, offering insights into liver biology, regeneration, and disease progression. The impact of this protocol extends beyond liver studies, potentially benefiting other autofluorescent tissues, making it a valuable tool for a wide range of research applications.

## General solutions and reagents

- Phosphate-buffered saline (PBS)
- Tris-buffered saline (TBS)
- 4% Paraformaldehyde (PFA) (Vwr, 20910.363) in PBS for fixation
- Ethanol (50%, 70%)
- TBS with 0.1% Triton X-100 (Sigma Aldrich, T8787) for membrane permeabilization
- Blocking solution: 4% Bovine Serum Albumin (BSA) (Merck, 126575) in TBS
- IF wash buffer: 0.01% Triton X-100, 0.025% Tween 20 (Sigma Aldrich, P1379), 0.05% BSA, pH 7.5 in TBS
- DAPI nuclear stain (Sigma-Aldrich, D9542) at 1:1000 dilution in blocking solution
- Sudan Black B (Sigma Aldrich, 199664) (1%) in 70% ethanol or True Black (Cell signaling, 92401S) for autofluorescence quenching
- Mounting medium Mowiol (Merck/Sigma Aldrich, 324590), Vectashield (Vector Laboratories, H-1000-10), or ProLong Gold (Thermo Fisher, P36930)
- Polypropylene 1,5 tubes (15386548, Fisher)
- 4′,6-diamidino-2-phenylindole (DAPI, D9542, Sigma-Aldrich, St Louis, MO, USA; dilution 1:1000).
- Primary antibodies (Typically diluted 1:100 in blocking solution)
  DppIV (R&D, AF954); GS (Sigma-Aldrich, G2781); HA-Tag (Bioke/CST, 3724S); HNF4a (Abcam, ab181604); Lyve1 (Abcam, ab14917).
- Secondary antibodies (Diluted 1:500 in blocking solution)
  Donkey anti-rabbit IgG Alexa Fluor^®^ 488 (Abcam, ab150073); Donkey anti-rabbit IgG Alexa Fluor^®^ 555 (Abcam, ab150074); Donkey anti-rabbit IgG Alexa Fluor^®^ 647 (Abcam, ab150075); Donkey anti-mouse IgG Alexa Fluor^®^ 555 (Thermo Fisher, A-31570); Donkey anti-mouse IgG Alexa Fluor^®^ 647 (Thermo Fisher, A-31571); Donkey anti-goat IgG Alexa Fluor^®^ 488 (Abcam, ab150129)
**Note:** If desired, PBS can replace TBS in all solutions. However, we standardly use TBS to avoid interference with phosphorylated antigens.

## Antigen retrieval buffer protocols

- **Glycine-HCl Buffer (pH 3.5)**
  - Dissolve 7.5 g of glycine in 1 L of distilled water.
  - Adjust pH to 3.5 with HCl.
- **Citrate Buffer (pH 6.0)**
  - Dissolve 2.1 g of citric acid monohydrate in 1 L of distilled water.
  - Adjust pH to 6.0 with NaOH.
- **EDTA Buffer (pH 8.0)**
  - Dissolve 0.37 g of EDTA in 1 L of distilled water.
  - Adjust pH to 8.0 with NaOH.
- **Tris-EDTA Buffer (pH 9.0)**
  - Dissolve 1.21 g of Tris base and 0.37 g of EDTA in 1 L of distilled water.
  - Adjust pH to 9.0 with HCl.

## Equipment

- Vibratome for tissue sectioning (Leica-VT 1000S Vibratome, Leica Biosystems, Nussloch, Germany)
- Confocal microscope with fluorescence capabilities (Olympus FV1000)
- Laboratory shaker
- Incubator capable of maintaining 95°C, or Microwave or Pressure cooker

## Methods

### Tissue preparation

1. Fix livers in 4% PFA for 48 h at 4°C to preserve tissue structure and protein epitopes.
2. Rinse fixed tissues three times with TBS (or PBS) for 15 min to remove residual PFA.
3. Transfer tissues to 70% ethanol for long-term storage at room temperature.
  IMPORTANT: When working with phospho-specific antibodies, it is often recommended to use TBS rather than PBS. TBS does not contain phosphate ions and is less likely to interfere with phospho-specific detection.

### Agarose embedding and vibratome sectioning

**Figure 1.**
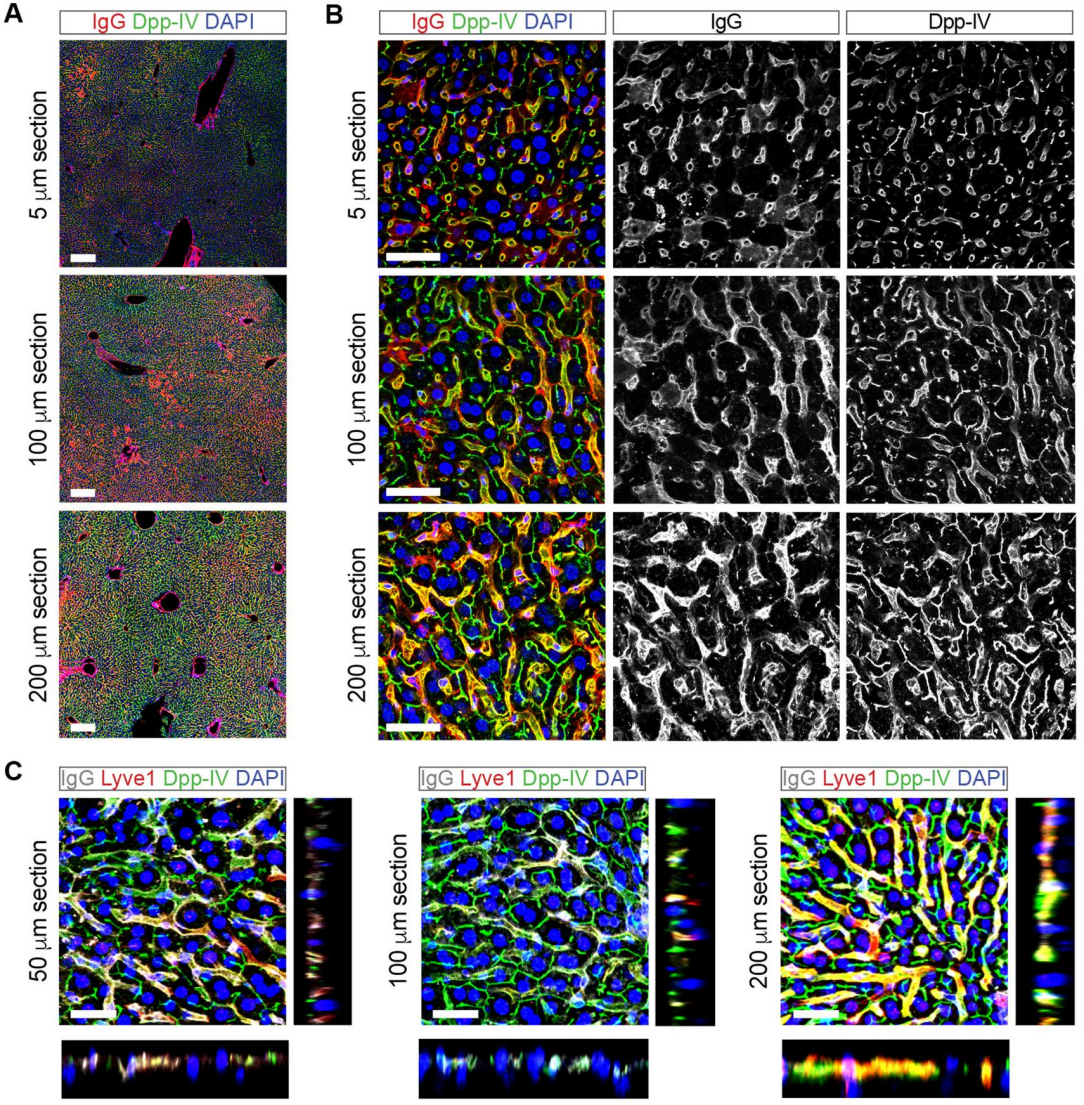
Comparison of sinusoidal and canalicular network visualization in thin vs. thick liver sections. (**A**) Low magnification images of thin sections (5 μm) from paraffin-embedded livers cut with a microtome and of thick sections (100 and 200 μm) from agarose-embedded and vibratome-cut livers. Anti-dipeptidyl peptidase-4 (Dpp-IV or CD26) antibody (green) marks the sinusoidal and canaliculi networks. Mouse immunoglobulins (IgGs) conjugated with Alexa-Fluor-555 (red) non-specifically detect endothelial and dead cells in mouse tissues [4, 19, 20]. Scale bar 250 μm. (**B**) High-magnification images of liver sections showing increased complexity of sinusoidal and canaliculi networks in thicker sections when compared to thin sections 5 μm. Scale bar 50 μm. (**C**) High-magnification images of thick vibratome-cut liver sections and orthogonal views of projections generated using Fiji show the complexity of the imaged tissue and the depth of antibody and light penetration. Anti-dipeptidyl peptidase-4 (Dpp-IV or CD26) antibody (green) marks the sinusoidal and canaliculi networks. Mouse immunoglobulins conjugated with Alexa-Fluor-647 (grey) mark all endothelial cells and show the sinusoidal network. Anti-Lyve1 antibody (red) marking sinusoids. Liver autofluorescence was quenched with Sudan Black B in all panels. Scale bar 50 μm

Cut 100 to 200 µm thick sections of liver tissue using a vibratome (Fig. 1). These thicker sections provide a more comprehensive 3D perspective, allowing detailed analysis of liver structures, such as hepatocytes, sinusoids, and bile canaliculi.

1. Rehydrate livers in 50% ethanol for 10 min, followed by TBS (or PBS) for 10 min.
2. Prepare 4% agarose (A9539, Sigma Aldrich) in TBS (or PBS) and microwave until agarose is in solution. Take care that the solution does not bubble over—take it out of the microwave periodically and swirl to mix. Keep solution in a 55°C water bath until ready to use.
3. Using forceps, place one median lobe at the bottom of a six-well plate. Only place one lobe per well. Fill each well to cover the liver lobe. Position the median lobe for sectioning in the appropriate plane. Avoid creating air bubbles around the liver, as this compromises the structural integrity of the agarose block during sectioning.
4. Agarose will set at room temperature within 30 min. If needed, transfer the six-well plate containing embedded liver lobes to 4°C until ready to section. If sectioning the next day, add TBS (or PBS) to cover the agarose in the well and seal the plate with parafilm. Store in a box containing moist paper towels soaked in TBS (or PBS) at 4°C.
5. Using a razor blade, cut out an agarose block containing the liver lobe. Make sure that the planes of section are straight and parallel to the sides of the tissue. One plane will serve as the surface on which the agarose block is glued down. Since the agarose does not infiltrate the tissue, do not trim too much agarose from around the tissue so that the tissue will be well supported.
6. Use cyanoacrylate glue to attach the block to the cutting surface.
7. Section at 50–200 μm thickness by vibratome. Sections can be transferred to 70% ethanol for long-term storage at room temperature or directly used for downstream steps.
8. Use forceps to dissect away the agarose from the sectioned tissue. Agarose will not penetrate the tissue but will melt and stick to the tissue during antigen retrieval steps.
9. Place the selected sections in 1.5 ml tubes filled with TBS and proceed to the following steps.

### Rehydration and antigen retrieval

####  

**Figure 2.**
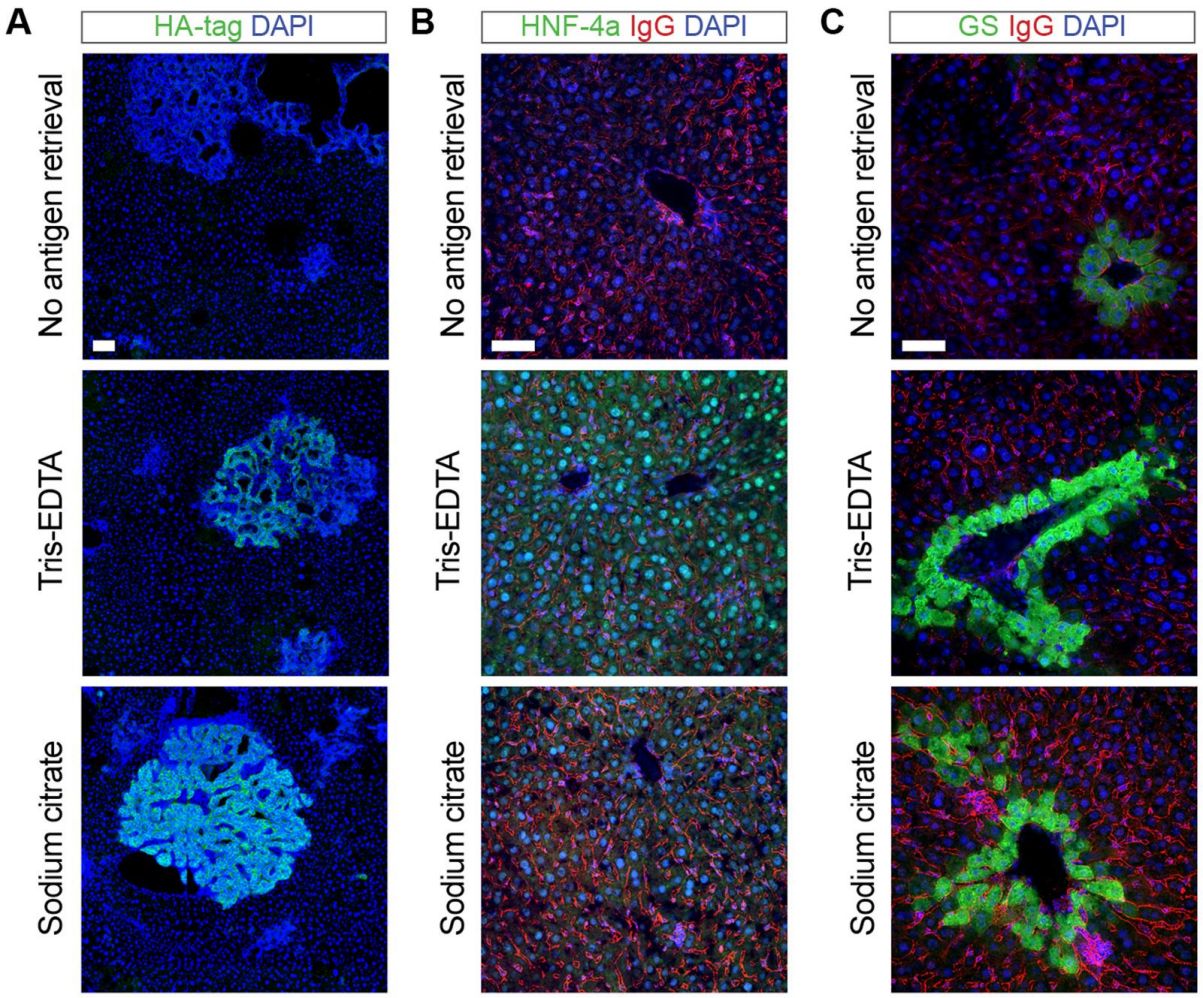
Comparison of different antigen retrieval methods on antibody efficiency. (**A**) Immunofluorescent staining of HA-tag-expressing cholangiocarcinoma tumors induced by the hydrodynamic injection of Sleeping Beauty vectors expressing the intracellular domain of Notch (NICD) and HA-tagged and myristoylated AKT [5]. Immunodetection of HA-tag (green) shows a stronger signal when the sections are pretreated with the Sodium Citrate antigen retrieval. Scale bar 100 μm. (**B**) Liver section showing the expression of HNF4-α (green) and mouse IgG (red). Immunodetection of HNF-4α shows a stronger signal when the sections are pretreated with Tris-EDTA antigen retrieval. Mouse IgGs non-specifically detect endothelial and dead cells in mouse tissues [4, 19, 20]. Scale bar 50 μm. (**C**) Liver section showing the expression of Glutamine synthetase (GS, green). While the immunodetection of GS works with and without antigen retrieval, the signal is stronger when the sections are pretreated with Tris-EDTA or with Sodium Citrate antigen retrievals. Scale bar 50 μm. Livers shown in (A–C) were sectioned at 100 μm. Liver autofluorescence was quenched with Sudan Black B in all panels. Scale bar 50 μm

This protocol covers antigen retrieval for immunofluorescence (or immunohistochemistry), using four different buffer options tailored to specific antigen requirements. While some retrieval buffers are commonly associated with certain antigen types or cellular localizations, there is no absolute correlation between retrieval buffer selection and protein type or localization [8–10]. Thus, each antibody requires optimization for antigen retrieval, as different retrieval buffers impact epitope exposure differently [8–10]. We suggest routinely testing all antigen retrieval buffers to determine the optimal conditions for each new antibody. In addition, the microwave, pressure cooker, and water bath methods can have different efficiencies depending on the antigen, tissue type, and antibody used. For instance, while the pressure cooker method is often preferred for highly crosslinked proteins that are difficult to unmask, it might be too destructive for some epitopes. In contrast, the water bath method is often considered as a milder but more broadly applicable method. Therefore, these methods also need to be tested on an antibody-by-antibody basis as they might vary in their ability to unmask antigens, influencing the outcome of immunostaining (Fig. 2).

1. Preheat the chosen buffer in a microwave-safe jar or staining container.
2. Rehydrate the sections if stored in ethanol. For this, incubate in 50% ethanol for 10 min, followed by TBS (or PBS) for 10 min.
3. Perform antigen retrieval based on the requirements of each primary antibody (needs to be experimentally tested):
  - Option 1: Glycine-HCl Buffer, pH 3.5, at 95°C for 20 min.
  - Option 2: Sodium citrate buffer, pH 6.0, at 95°C for 20 min.
  - Option 3: EDTA Buffer, pH 8.0, at 95°C for 20 min.
  - Option 4: Tris-EDTA buffer, pH 9.0, at 95°C for 20 min.
  - Option 5: No antigen retrieval (proceed directly to blocking steps).
4. Antigen Retrieval Methods:
  - Microwave Method:
    - Place tissue section in 1.5 ml tubes filled with the preheated buffer of choice.
    - Heat in the microwave at medium power until a gentle boil is reached.
    - Once boiling, reduce power slightly and continue heating to maintain a steady boil for 10–20 min.
  - Pressure Cooker Method:
    - Place tissue sections in 1.5 ml tubes filled with the buffer of choice within the pressure cooker.
    - Seal and bring to full pressure, maintaining heat for 2–5 min.
    - Allow the cooker to cool down and release pressure before opening.
**Note:** Do not close the cap of the tube tightly, and make sure to use centrifuge tubes made of 100% virgin polypropylene (15386548, Fisher), which are autoclavable and safe to use in a pressure cooker.
  - Water Bath Method:
    - Preheat the buffer of choice in a water bath to 95°C.
    - Place tissue sections in 1.5 ml tubes filled with the buffer and incubate for 20–30 min at this temperature.
5. Cooling and Washing:
  - Allow slides to cool in the buffer at room temperature for 30 min.
    IMPORTANT: Allow sections to gradually cool to room temperature: It is very important that cooling down is not done abruptly, as this will promote the folding of proteins and may hide relevant epitopes. Always allow sections to cool down slowly.
  - Rinse sections twice in PBS (or TBS) for 10 min to remove excess buffer, preparing them for blocking and antibody incubation.

### Blocking and antibody incubation

1. Incubate sections in blocking solution (4% BSA in TBS) for 2 h on a shaker to reduce nonspecific binding.
2. Place up to five sections in individual 1.5 ml tubes filled with TBS. Each tube should correspond to one antibody combination. Use more tubes if several antibody combinations will be performed.
3. Dilute the primary antibody in blocking solution. While we typically use a dilution of 1:100 of antibody in blocking solution for most antibodies, the ideal antibody dilution may need to be empirically validated on an antibody-by-antibody basis (dilution range: 1:50–1:2,000).
4. Incubate sections overnight with the primary antibody solution at room temperature on a shaker. 100 µl of primary antibody solution is typically sufficient to cover up to five liver sections in a 1.5 ml tube, but the volume needs to be adapted if the number or size of the sections increases.
**Note 1:** Normal serum might be used as an alternative to BSA to block non-specific protein binding in tissues with strong Fc receptor binding.
**Note 2:** Antibody penetration for the detection of nuclear antigens can be enhanced by performing a permeabilization step using TBS with 0.1% Triton X-100 for 10 min, followed by two washes with TBS prior to the incubation with 4% BSA in TBS.
**Note 3:** Overnight incubation of primary antibody is typically sufficient to ensure thorough penetration of antibodies in the tissue, but incubation times for thick sections might need to be empirically determined as thicker sections might require longer incubation periods.

### Washing and secondary antibody incubation

1. Wash sections three times with IF wash buffer for 10 min each.
2. Dilute secondary antibody (Alexa Fluor^®^ 488 -ab150073- or with Alexa Fluor^®^ 555 -ab150074-. Abcam, Cambridge, MA, USA) at a dilution of 1:500 in blocking solution and add 4′,6-diamidino-2-phenylindole (DAPI. D9542, Sigma-Aldrich, St Louis, MO, USA. Dilution 1:1000). Alternatively, mouse blood vessels can be detected non-specifically by incubation with Donkey anti-mouse IgG (Alexa Fluor^®^ 488 ab150073 or Alexa Fluor^®^ 555 ab150074, Abcam, Cambridge, MA, USA).
3. Incubate sections with the secondary antibody solution for 2 h at room temperature and protect from light.

### Sudan black staining for autofluorescence quenching

**Figure 3.**
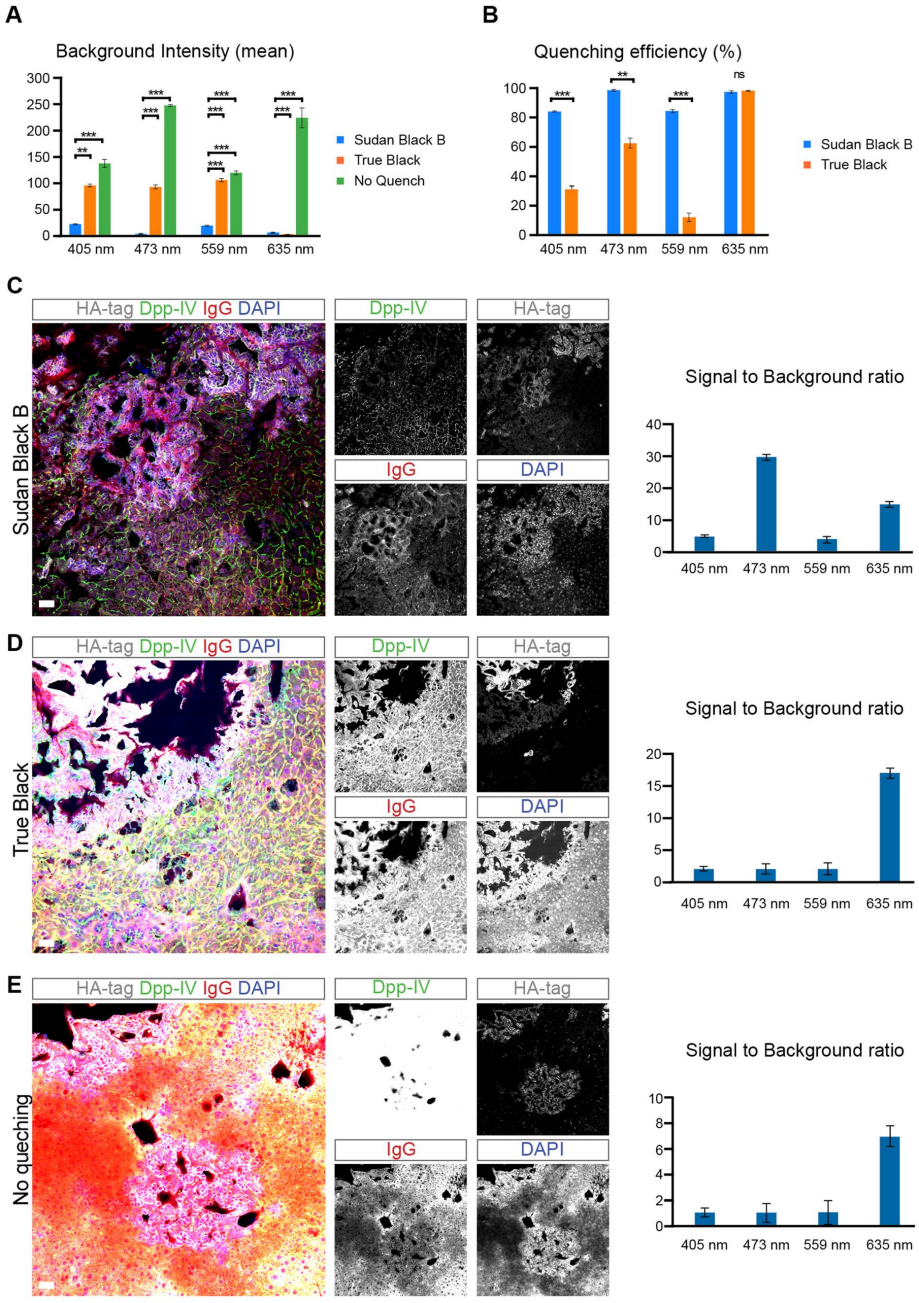
Liver autofluorescence quenching with Sudan Black B. Autofluorescence in liver tissue was measured by stimulating the sample (liver sections) using excitation at 405, 473, 559, and 635 nm. Emission was collected using filters corresponding to 567, 640, and 655–755 nm. Autofluorescence intensity was quantified across these channels to evaluate potential spectral overlap with fluorophores used in the study (DAPI, Alexa Fluor 488, Alexa Fluor 647, and Cy3). (**A**) Background intensity (mean) of endogenous liver autofluorescence and (**B**) Quenching efficiency in percentage from unquenched liver sections and compared with quenched liver sections with Sudan Black B or True Black/Percentage of quenching efficiency of liver sections quenched with Sudan Black B or True Black and this in comparison to unquenched liver sections (**C-E**) Immunofluorescent staining (on the left) of HA-tag-expressing cholangiocarcinoma tumors induced by the hydrodynamic injection of Sleeping Beauty vectors expressing the intracellular domain of Notch (NICD) and HA-tagged myristoylated AKT [5, 21]. Ha-tag antibody (grey) marks tumor cells driven by the expression of HA-tagged AKT. Anti-dipeptidyl peptidase-4 (Dpp-IV or CD26) antibody (green) stains the sinusoidal and canaliculi networks. Mouse immunoglobulins conjugated with Alexa-Fluor-555 (red) non-specifically bind to endothelial and dead cells in mouse tissues [4, 19, 20]. Liver sections were incubated with Sudan Black B (C), True Black (D), or without any quenching (E). The signal-to-background ratio of each channel (on the right), which shows the efficacy of the different auto fluorescent quenching methods. Livers shown were sectioned at 100 μm. Scale bars 50 μm

**Figure 4.**
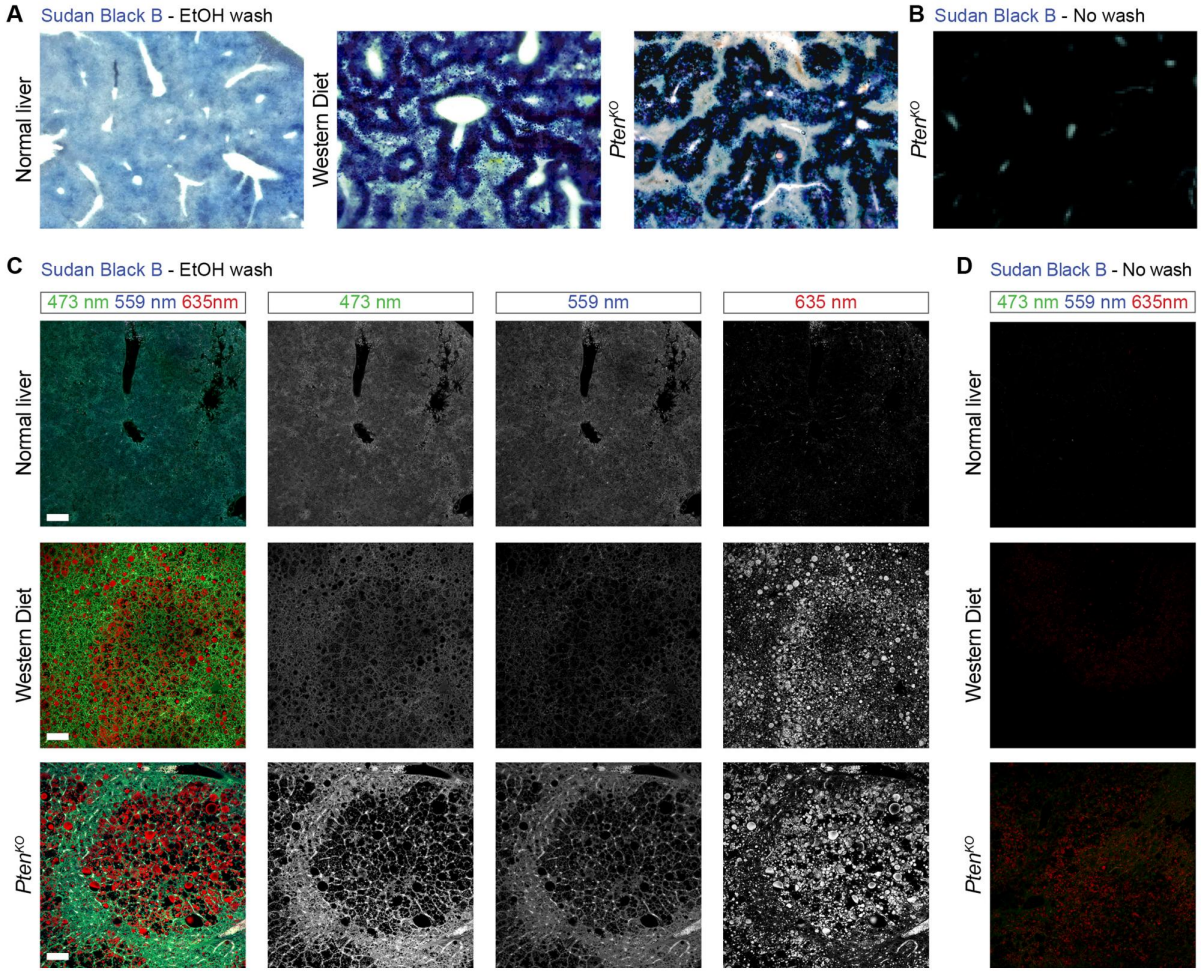
Lipid droplet detection in steatotic livers with Sudan Black B. (**A**) Comparison of bright field images from normal and steatotic mouse livers stained with Sudan Black B and washed with 70% EtOH to remove the excess of Sudan Black B while retaining the staining bound to lipid droplets. Lipid droplet detection with Sudan Black B shown in normal livers and in steatotic livers from two models of fatty liver disease induced either by 8 weeks of ad libitum feeding of Western diet or by the deletion of *Pten* in mouse hepatocytes [16, 17]. Deletion of *Pten* in hepatocytes was obtained by injecting an adeno-associated virus 8 that expresses Cre from a hepatocyte specific promoter (AAV-TGB-CRE) into 8-week-old *Pten^fl/fl^* mice. (**B**) Bright field picture of a steatotic *Pten* mutant stained with Sudan Black B without post-staining wash with 70% EtOH; showing the liver parenchyma completely stained without distinction of steatotic and non-steatotic cells. (**C**) Immunofluorescent images showing the detection of lipid droplets by Sudan Black B autofluorescence (635 nm) in steatotic livers (Western diet and *Pten* mutant) after 70% EtOH washes to remove staining from normal parenchyma while maintaining it in lipid droplets. The other channels show liver autofluorescence in normal and steatotic livers. Scale bar 250 μm. (**D**) Immunofluorescent images showing the lack of autofluorescence or lipid detection in normal and steatotic livers stained with Sudan Black B but without washes in 70% EtOH

1. Wash sections three times with IF wash buffer for 10 min each.
2. Place sections in 50% ethanol for 10 min to prepare for Sudan Black B treatment.
3. Incubate sections in 1% Sudan Black B (Sigma Aldrich, 199664) solution in 70% ethanol for 20 min. Sudan Black B binds to lipofuscins [11–15], effectively quenching autofluorescence. Make sure that all tissues are properly covered in Sudan Black B solution and that different tissue sections do not remain in contact, as any of these options will lead to suboptimal autofluorescence quenching.
**Note 1:** True Black is another option to quench liver autofluorescence, but, given that its quenching efficiency is lower to Sudan Black B, the right acquisition settings need to be applied for proper imaging. Nonetheless, we recommend using Sudan Black B as we find that Sudan Black B is more efficient than True Black in quenching liver autofluorescence (Fig. 3).
**Note 2:** Besides quenching tissue autofluorescence for the detection of fluorescent markers, Sudan Black B can also be used as an option to detect lipid droplets (Fig. 4). However, simply incubating liver tissues in Sudan Black B masks lipid droplets because the whole liver parenchyma will stain regardless of the lipid content. Therefore, after incubating the sections with Sudan Black B, two additional washes of 70% EtOH for 10 min each are required to retain the Sudan Black B bound to lipid droplets while removing the Sudan Black B bound to lipofuscins present in normal tissues. Figure 4 shows lipid droplet detection with Sudan Black B in steatotic livers from two models of fatty liver disease induced either by 8 weeks of ad libitum feeding of the Western diet or by the genetic deletion of *Pten* in mouse hepatocytes [16, 17].

### Mounting

1. Rinse sections briefly in TBS (or PBS) to remove excess of Sudan Black B.
2. Mount sections on a glass slide without transferring any liquid. Add sufficient mounting medium, such as Mowiol, to cover all sections and place a coverslip on top. Apply mild pressure to remove the excess of mounting medium and allow the Mowiol to dry in the dark and at room temperature for 30 min before proceeding to imaging. Alternatively, other mounting media, such as Vectashield or Prolong Gold can be also used following the manufacturer’s instructions.

### Imaging

**1. Image Adquisition by Confocal Microscopy **
Adjust laser power and detector sensitivity to prevent photobleaching and optimize signal-to-noise ratio.
  Images were acquired using an Olympus Fluoview FV1000 confocal microscope system equipped with a UPLSAPO 20x objective (NA: 0.75). The pinhole diameter was 85um. Images were captured at 1024x1024 pixels resolution with 16-bit depth. The excitation wavelengths and emission filters for each channel are shown in Table 1.
**2. Autofluorescence Quenching and Signal-to-Noise Ratio Analysis**
  Liver autofluorescence was stimulated using excitation at 405, 488, 555, and 640 nm. Emission was collected using filters corresponding to 567 nm (CH1 and CH4), a dichroic mirror at 640 nm (CH2), and a 655–755 nm bandpass filter (CH3). Autofluorescence intensity was quantified across these channels to evaluate potential spectral overlap with fluorophores used in the study (DAPI, Alexa Fluor 488, Alexa Fluor 647, and Cy3). Autofluorescence intensity was quantified in each detection channel to assess its potential overlap with specific fluorophore signals.
  Autofluorescence intensity and quenching were quantified using Fiji (ImageJ). Fluorescence images were split into individual channels corresponding to Alexa 488, Cy3, Alexa 680, and DAPI using the “Split Channels” function. Regions of interest (ROIs) were selected in representative tissue areas, and mean fluorescence intensity was measured using “Analyze > Measure”. To assess autofluorescence quenching, intensity values from samples treated with Sudan Black B and True Black were compared to untreated controls, and quenching efficiency was calculated as
  $$\mathrm{Quenching}\;\left(\%\right)=\left(1-\frac{\mathrm{Treated}\;\mathrm{mean}\;\mathrm{intensity}}{\mathrm{Untreated}\;\mathrm{mean}\;\mathrm{intensity}}\right)x\;100$$

**Table 1.** Imaging parameters for multichannel fluorescence detection.

Channel	Excitation (nm)	Detector voltage (PMT)	Dye name	Emission filter
CH1	405	517 V	DAPI	567 nm
CH2	473	472 V	Alexa Fluor 488	SDM640 (Emission DM)
CH3	635	602 V	Alexa Fluor 647	BA655-755 (Emission Bandpass 655–755 nm)
CH4	559	517 V	Cy3	Mirror (Emission 567 nm)

Signal-to-noise ratio (SNR) was determined by measuring mean fluorescence intensity (m signal) within the tissue ROI and the standard deviation of background fluorescence (s background) from an ROI in a non-signal region. SNR was calculated as:


$$\mathrm{SNR}=\frac{\mu \;\mathrm{signal}}{\sigma \;\mathrm{background}}$$

#### Overview of the technique and expected outcomes

This protocol introduces key advancements in liver immunofluorescence staining. By employing 100–200 µm vibratome-cut sections it facilitates detailed 3D-like analysis of liver architecture, including hepatocytes, sinusoids, and bile canaliculi, which are often lost in traditional 5 µm paraffin sections (Fig. 1A and B). The protocol incorporates optimized antigen retrieval steps, using tailored buffers at different pH levels (e.g. Citrate Buffer pH 6.0, Tris-EDTA pH 9.0, EDTA pH 8.0, Glycine-HCl pH 3.5), to maximize epitope exposure and ensure compatibility across diverse antibodies (Figure 2). Additionally, to address the challenge of autofluorescence from lipofuscin, vitamin A, and lipids, Sudan black staining is applied, effectively quenching background signals and significantly enhancing signal clarity (Figure 4).

These methodological improvements yield high-resolution images with minimal autofluorescence interference, enabling precise localization of target proteins and comprehensive visualization of liver microarchitecture. The use of thick vibratome sections supports a 3D-like analysis of complex structures, such as hepatocyte arrangements and bile canaliculi networks, while Sudan Black B treatment enhances signal specificity. Together, these optimizations provide a robust and reproducible framework for structural and molecular studies of liver tissue, offering significant value to researchers investigating liver regeneration, cancer, and chronic disease pathology.

#### Advantages and limitations

The optimized immunofluorescence protocol offers a simple approach to study liver tissue by providing a 3D-like perspective that enhances the visualization of liver architecture and precise marker localization. By effectively quenching liver autofluorescence, it ensures significantly improved signal clarity, enabling more accurate fluorescence-based studies. Tailored antigen retrieval steps further enhance antibody compatibility and signal quality, supporting versatile applications across various markers and epitopes. Although the method requires specialized equipment, such as a vibratome for thick tissue sectioning, this equipment is relatively affordable, easy to use, and allows for rapid processing. However, challenges arise when using antibodies with incompatible antigen retrieval protocols, particularly when attempting to simultaneously detect multiple markers. Despite these limitations, the protocol remains a powerful tool for detailed structural and molecular analysis of liver tissue.

#### Troubleshooting

To address common issues encountered during liver immunofluorescence staining, several troubleshooting strategies can be applied (Table 2). The liver is composed of relatively large cells (hepatocytes) and complex networks of sinusoids, canaliculi, and bile ducts, which is solved by using thick vibratome sections (50–200 µm) rather than thin paraffin sections (5–7 µm). However, imaging thick tissue sections (100–200 µm) can be limited by the microscopy system used due to light penetration [18]. Confocal microscopy typically allows visualization up to 30–100 µm in liver tissue due to light scattering and absorption. In contrast, two-photon microscopy (200–500 µm) and light-sheet microscopy (up to 800 µm) provide deeper tissue penetration, making them better suited for thick-section imaging [18]. If deeper visualization is needed, alternative imaging modalities should be considered.

**Table 2.** Common imaging issues and recommended solutions.

Observed issue	Possible solution
Weak signal	Optimize antigen retrieval
	Increase incubation time of primary antibody
	Include 0.1%triton-TBS permeabilization step if the epitope is nuclear
High background	Increase Sudan Black concentration
	Increase blocking time
Inconsistent staining	Ensure uniform section thickness
	Ensure through rehydration of tissue section
Low light penetration	Replace confocal imaging by Two-Photon or Light sheet microscopy.

Often, nuclear epitopes require an additional incubation step of 10 min with 0.1% Triton X-100 in TBS to permeabilize the nuclear envelope and cell membrane, which facilitates the penetrance of antibodies to the nucleus. This step, however, can destroy sensitive epitopes located on the cell membrane, so it needs to be optimized on an antibody-by-antibody basis. High background fluorescence can be mitigated by increasing the blocking time or adjusting the concentration of Sudan Black B. Additionally, ensuring that antigen retrieval protocols are properly tailored for each antibody is crucial for reducing unwanted background signals. If weak signal intensity is observed, changing antibody concentrations or extending the primary antibody incubation time may enhance signal strength. Additionally, optimizing antigen retrieval time is an essential step.

Inconsistent staining across sections can often be attributed to variations in section thickness or improper rehydration. Thick sections can pose a diffusion barrier that impedes antibody penetration. Prolonged incubation times with gentle rotation, permeabilization, heat or increased pressure can enhance antibody penetration. Ensuring uniform section thickness during vibratome cutting and thorough rehydration of tissue sections is critical for maintaining staining consistency. Moreover, fine-tuning antigen retrieval conditions specific to each antibody can help achieve reliable and reproducible staining results. These adjustments ensure the robustness and precision of the protocol, enabling high-quality fluorescence imaging of liver tissue.

#### Applications and target audience

This protocol is particularly suited for researchers focused on liver regeneration, disease pathology, and structural analysis, enabling high-resolution imaging of liver microarchitecture. However, researchers interested in exploring complex tissue structures in other autofluorescent tissues, such as the kidney, brain, pancreas, spleen, and adipose tissue, will find this method beneficial [12–15]. These share similar autofluorescent components, such as lipofuscin, and lipid deposits, that interfere with traditional fluorescence imaging. This optimized protocol can enhance the clarity and depth of fluorescence imaging across these tissues, providing a more accurate and detailed analysis of cellular and tissue structures.
